# Informed classification of sweeteners/bitterants compounds via explainable machine learning

**DOI:** 10.1016/j.crfs.2022.11.014

**Published:** 2022-11-14

**Authors:** Gabriele Maroni, Lorenzo Pallante, Giacomo Di Benedetto, Marco A. Deriu, Dario Piga, Gianvito Grasso

**Affiliations:** a Dalle Molle Institute for Artificial Intelligence IDSIA - USI/SUPSI, Via La Santa 1, CH-6962, Lugano-Viganello, Switzerland; b Polito^BIO^MedLab, Department of Mechanical and Aerospace Engineering, Politecnico di Torino, Corso Duca degli Abruzzi 24, 10129, Torino, Italy; c 7HC srl, Via Giovanni Paisiello 55, 00198, Rome, Italy

**Keywords:** Sweet/bitter dichotomy, Explainable machine learning, Natural compounds, Sweetener, Bitterants

## Abstract

Perception of taste is an emergent phenomenon arising from complex molecular interactions between chemical compounds and specific taste receptors. Among all the taste perceptions, the dichotomy of sweet and bitter tastes has been the subject of several machine learning studies for classification purposes. While previous studies have provided accurate sweeteners/bitterants classifiers, there is ample scope to enhance these models by enriching the understanding of the molecular basis of bitter-sweet tastes. Towards these goals, our study focuses on the development and testing of several machine learning strategies coupled with the novel SHapley Additive exPlanations (SHAP) for a rational sweetness/bitterness classification. This allows the identification of the chemical descriptors of interest by allowing a more informed approach toward the rational design and screening of sweeteners/bitterants. To support future research in this field, we make all datasets and machine learning models publicly available and present an easy-to-use code for bitter-sweet taste prediction.

## Highlights

• Machine learning model for the classification of sweeteners/bitterants compounds.
• Informed approach toward the rational screening of sweeteners/bitterants.
• Implementation of an easy-to-use code for bitter-sweet taste prediction.

## Graphical abstract

**Figure:**
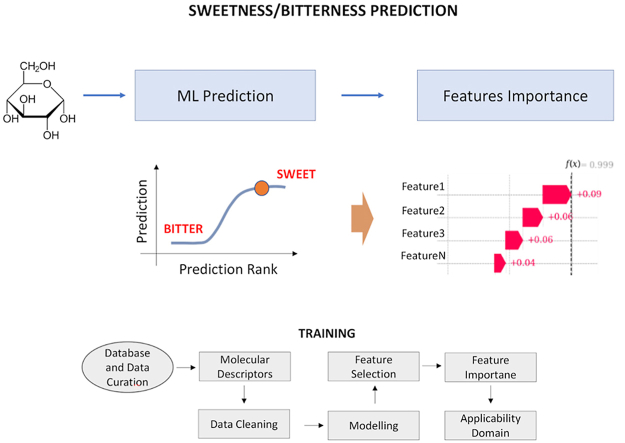

## 1 Introduction

Bitter and sweet tastes along with umami, saltiness, and acidity represent the fundamental taste senses (Besnard et al., 2016), which are linked to specific biological and survival needs. For example, the bitter taste has evolved to protect organisms from the consumption of potentially poisonous substances, whereas the sweet taste is normally associated with the energetic and caloric content of foods. Both sweet and bitter molecules are recognized by G-protein coupled receptors (GPCR), but while taste receptors type 2 (TAS2Rs) are primarily responsible for detecting bitter tastants, the TAS1R2/TAS1R3 heterodimer belonging to class-C GPCR is known to be involved in the sensation of sweetness (Li et al., 2002). These receptors are located on apical membranes of taste receptor cells located in the taste buds. Human gustatory systems are characterized by the dichotomy between sweet and bitter tastes with an innate preference for sweet tastes and an aversion to bitter tastes. The sensation of bitter-sweet taste is an emerging property arising from complex molecular interactions of a compound with these receptors. Besides the oral cavity, taste receptors are also present in other body parts such as the urethra (Deckmann et al., 2014), skin (Ho et al., 2021; Shaw et al., 2018), brain (Singh et al., 2011), heart (Foster et al., 2013, 2014), and pancreas (Kyriazis et al., 2012, 2014). As well as their primary role in taste perception (in the oral cavity), such receptors are also implicated in diabetes and obesity by virtue of their roles in nutrient perception, glucose level maintenance, appetite regulation, as well as hormone release (Behrens and Lang, 2022).

As a food additive for a long time, sweeteners have been widely used in the food industry (Carocho et al., 2017). There are lots of controversies and challenges relating to the sweetener industry in recent years, though improvements in technologies have greatly accelerated its development. When developing sweeteners, not only do they need to taste sweet, but they also need to have no harmful side effects, which increased the demand for the development of new sweeteners in the food industry. Within this framework, finding compounds with a pleasant gradient of bitter-sweet flavor may lead to the development of low-calorie sweeteners and bitter masking molecules.

The design and development pipeline of sweeteners usually follows the following pathway: extraction, separation, and identification of potential molecules from natural plants and synthesis. The previously-mentioned procedures are highly expensive and require complex chemical or biological characterization of the samples. Within this view, it is clear that computational prediction and simulation of potential compounds in the early stage could accelerate the design and development process of sweetener molecules (Bouysset et al., 2020).

In silico methodological approaches for the bitterant prediction include structure-based, ligand-based and machine-learning methods (Bahia et al., 2018; Malavolta et al., 2022); of particular interest for the present study are these latter approaches. Naive Bayes approach and circular fingerprint have been carried out in literature to classify bitterness by using a dataset of about 600 bitterants taken from a proprietary database and more than 10,000 non-bitterants randomly selected from the MDL Drug Data Repository (MDDR) (Rodgers et al., 2006). The model was characterized by accuracy, precision, specificity, and sensitivity of 88, 24, 89, and 72% respectively in the five-fold cross-validation. Although the previously mentioned study reports the first bitterant prediction algorithm based on a quite large dataset, the work didn't provide a prediction tool that can be used by users to test their molecules. Huang et al. addressed this issue by developing the first online toolkit of bitterness prediction called “BitterX”. The web application uses a Support Vector Machine (SVM) approach (Vapnik, 1995) on physicochemical descriptors (Huang et al., 2016). In their study, the dataset is composed of 539 publicly available bitterants and 539 non-bitterants taken from the Available Chemicals Directory (ACD) database. The computational model offers remarkable accuracy and precision of more than 91% and sensitivity within the range of 91–94% on the test set. However, several small molecules considered as the non-bitterants are still not experimentally tested. The adaptive ensemble machine-learning method “Adaptive Boosting” (AdaBoost) was applied in another study to build a bitterness classifier called “BitterPredict” (Dagan-Wiener et al., 2017). The model was trained on 12 basic physicochemical descriptors and 47 Schrödinger QikProp descriptors (Dagan-Wiener et al., 2017). The BitterDB (Dagan-Wiener et al., 2019; Wiener et al., 2012), in combination with the data from Rojas et al. (2016) was used to identify bitterants, while most of the non-bitterants (1360 non-bitter flavours) were still hypothetical (Zheng et al., 2018). The prediction model gives the accuracy (83%), precision (66%), specificity (86%), and sensitivity (77%) on the test set. Recently, the consensus voting strategy based on multiple ML models has been used in literature to perform the bitterant classification task considering a dataset of experimentally confirmed bitterants and non-bitterants (Zheng et al., 2018).

Regarding the bitter/sweet dichotomy and the in-silico taste prediction, three major examples have been recently published, i.e., BitterSweetForest (Banerjee and Preissner, 2018), BitterSweet (Tuwani et al., 2019) and VirtualTaste (Fritz et al., 2021). BitterSweetForest and VirtualTaste are based on the random forest classification algorithm and Morgan molecular fingerprints, whereas BitterSweet is based on Dragon molecular descriptors and relies on the Adaboost method. BitterSweetForest was able to reach incredibly high predictive performance (e.g., AUROC of 0.98 both in cross-validation and external validation), but with a relatively low number of compounds in the dataset (517 artificial and natural sweet compounds and 685 bitter molecules). On the other hand, BitterSweet remarkably enlarged the bitter/sweet dataset collecting positive sets of 813 bitter and 1139 sweet molecules but achieving lower performance compared to BitterSweetForest. Virtual Taste extends the previous work of the same authors, BitterSweet Forest, with a richer dataset and develops three models based on the random forest algorithm, Morgan molecular fingerprints and different data sampling methods for bitter/non-bitter prediction (VirtualBitter model achieving AUROC values of cross-validation and external validation of 0.97 and 0.96, respectively), sweet/non-sweet prediction (VirtualSweet model achieving AUROC values of cross-validation and external validation of 0.97 and 0.96, respectively) and sour/non-sour prediction (VirtualSour model achieving AUROC values of cross-validation and external validation of 0.97 and 0.99, respectively). In VirtualTaste and BitterSweetForest the authors train different models on different families of descriptors so that the final subset of features is the one that provides the best performing model. Finally, to understand which features contribute the most to the change in the expected class a Bayesian-based feature analysis was employed in which the relative frequency of important features for each class was calculated taking the feature position and occurrence within the class and the relative feature frequency of that particular feature with respect to the other classes. In BitterSweet, on the other hand, a feature selection method and a feature compression method were compared, in the first relevant features for bitter-sweet prediction were identified using the Boruta algorithm (Kursa et al., 2010), in the second Principal Component Analysis (PCA) was used to reduce the dimensionality of the feature space. Finally, to explain the most impacting features of the model, a global feature ranking based on random forest relative feature importance with a mean decrease in Gini impurity was used.

The present study focuses on the development and testing of several machine learning strategies for sweetness/bitterness classification starting from the collection of compounds from several datasets available in the literature. Compound features were computed by using molecular descriptors from open-source libraries starting from the SMILES representations. The main contributions lie in the methods used for the feature selection and the interpretation of the resulting models. Previously discussed model explanation approaches based on random forest impurity-based feature importance provide only global interpretations in the form of feature relevance ranking for the model, furthermore they suffer from known disadvantages such as underestimation of the relative importance of features due to multicollinearity, and bias towards high cardinality features (Strobl et al., 2007). In this work, in order to improve the interpretability of the final model, we propose a method of sequential selection of relevant and uncorrelated or weakly correlated features based on hierarchical clustering on the feature's Spearman rank-order and two-sample Kolmogorov - Smirnov test. As a method of explanation, we propose to use the novel SHapley Additive exPlanations (SHAP) (Lundberg and Lee, 2017) approach which, in addition to having a solid mathematical background, provides a wider range of both global interpretation tools, such as feature importance graphs, summary and partial dependence plots, and local interpretation tools such as visualizations of the contribution of each single features in the bitter/sweet prediction of a single molecule in the dataset. This allows the identification of the chemical descriptors of interest by allowing a more informed approach to the design and screening of sweeteners/bitterants.

## 2 Materials and methods

### 2.1 Database and data curation

The employed dataset collects compounds from several previous pieces of literature. In particular, we gathered compounds from (i) Biochemical Targets of Plant Bioactive Compounds by Gideon Polya (2003), (ii) BitterDB (Wiener et al., 2012), (iii) Fenaroli Handbook of Flavor Ingredient (Burdock, 2016), (iv) DB by Rodgers et al. (2006), (v) DB by Rojas et al. (2017), (vi) SuperSweet (Ahmed et al., 2011), (vii) The Good Scents Company Database (http://www.thegoodscentscompany.com/), (vii) DB by Wiener et al. (Dagan-Wiener et al., 2017), (ix) SweetenersDB (Chéron et al., 2017). The resulted starting database collected a total of 3130 compounds (1764 sweet and 1366 bitter) with their SMILES description. We then checked all the SMILES using the RDKit library (http://www.rdkit.org), removing compounds with incorrect SMILES, searching for the relative correct SMILES in the PubChem database and removing duplicates. Then, the SMILES were processed with the ChEMBL Structure Pipeline (Bento et al., 2020) (https://github.com/chembl/ChEMBL_Structure_Pipeline) to highlight possible issues in the retrieved molecular structure and to standardize the SMILES representation for the entire dataset. The latter protocol runs a molecule checker on the compound structure, standardizes chemical structures and generates the parent molecule representation based on a set of predefined rules. At the end of this preprocessing pipeline, we obtain a final dataset of 2686 compounds (1415 sweet and 1271 bitter). A summary of the final collected compounds from each of the above-mentioned databases is reported in Table S1. It is worth mentioning that a similar approach to dataset creation was adopted in previous literature (Tuwani et al., 2019). We added some new compounds from new sources or updated version of the selected DBs. compared to the previous work, we increased the total number of compounds by 500, adding 168 sweet compounds and 355 bitter compounds.

### 2.2 Molecular descriptors

Starting from the SMILES representations, compound features were computed by using molecular descriptors from open-source libraries, i.e. RDkit (http://www.rdkit.org), pybel (O'Boyle et al., 2008) and Mordred (Moriwaki et al., 2018). In detail, we decided to focus on 2D molecular descriptors, using 208 descriptors from RDKit, 25 from pybel and 1826 from Mordred, obtaining a total of 2059 molecular features per molecule. We focused our attention only on the 2D molecular descriptors to avoid the impact of compound optimization and parameters related to the three-dimensional properties of molecules. The 2D descriptors provide fundamental chemical information in terms of molecular weight, number of individual types of atoms, types of bonds, degree of hybridization, spectral diameter, detour index, number of hydrogen donors and acceptors, molecular distance edge between different types of atoms, the polarizability of atoms and bonds, and topological polar surface. Moreover, other features derived from a symbolic representation were also considered such as the Zagreb index, adjacency matrix descriptors, Moreau–Mroto descriptors, Moran coefficients, Geary coefficients, and descriptors describing the Burden matrix and Barysz matrix (Czub et al., 2021). It is worth mentioning that other previous works successfully obtained good results in the field of taste prediction using only 2D molecular descriptors (Bouysset et al., 2020; Tuwani et al., 2019): this represents a great step forward since 2D molecular descriptors are less expensive from a computational point of view and not affected by variations in the three-dimensional molecular structures. However, 2D descriptors are not able to catch variations in the molecular three-dimensional arrangements of investigated molecules. This could be potentially important in the bitter/sweet taste prediction field, since some compounds can elicit both taste sensations depending on modifications in their 3D structural properties, including isomerism (Bachmanov et al., 2016, DuBois, 2016, Kawai et al., 2012, Naim et al., 1982, Schiffman et al., 1982, Shin et al., 1995, Temussi, 2012). Nevertheless, to avoid any possible misclassification for the above-mentioned type of compounds and employ only the 2D molecular descriptors, as also mentioned in the *Data Cleaning* section, we have not considered 70 compounds with identical 2D descriptors but different tastes. The inclusion of also 3D descriptors might be considered in the future to include compounds able to trigger sweet or bitter taste depending on their three-dimensional rearrangements.

### 2.3 Data cleaning

The resulting raw dataset, consisting of 2686 samples and 2060 columns (2059 features + 1 target column) was cleaned with the following procedures. First, 713 duplicate rows (or groups of more than 2 identical rows) were identified. 643 of them had the target variable duplicated, while the remaining 70 had a different target variable. Of the former, only one sample per group of duplicate rows was kept in the dataset, while the latter were entirely removed from the dataset to avoid ambiguity. Afterwards, all columns with a percentage of missing values greater than or equal to 95% have been removed from the dataset along with all columns with zero or almost zero variance, i.e., constant or near-constant columns such that for 99% or more of the samples the same numerical value is present in the dataset. Finally, all the columns with duplicate values (or groups with more than 2 identical columns) have been collapsed into a single column to avoid redundancy. Bitter and sweet classes have been replaced with the numeric values of 0 and 1, respectively. The cleaned dataset was thus reduced to 2195 samples and 1403 columns (1402 numerical features + 1 binary target column).

### 2.4 Validation strategies and evaluation criteria

Stratified 5-fold cross-validation was used for training and hyper-parameter tuning. Stratification allows for the preservation of the classes’ proportion in the created folds. Repeated 10-times 10-fold stratified cross-validation using different randomization of the data at each repetition was used for statistical comparison of modelling results and model selection. Models were evaluated using as primary evaluation criteria threshold-independent metrics such as Area Under Receiver Operating Characteristic Curve (AUROC) and Area Under Precision-Recall Curve (AUPRC), along with F1-score, Precision, and Recall.

### 2.5 Modelling

#### 2.5.1 We tested

· Two conventional statistical approaches, namely, a parametric logistic regression model and a non-parametric k-nearest neighbours algorithm;
· Two tree-based machine learning models, namely a random forest and a gradient boosting machine (LightGBM implementation);
· A deep learning model, i.e., multilayer perceptron (MLP).

A brief description of each model is provided in the following.

Logistic regression provides the probability of a certain class, where the log-odd is a linear combination of the input features. As a consequence, the class decision boundary is a linear function of the inputs. Linearity makes the estimation procedure simple and the results easy to understand and interpret. However, the correctness of the model depends on strong assumptions about the data including normality, independence, linearity and homoscedasticity. In a k-nearest neighbours classification algorithm, a new sample is assigned to the most common class among its k nearest neighbours. In random forest and gradient boosting machines, the prediction of the target variable is given as the result of an ensemble of weak models which are typically decision trees. A random forest fits several decision tree classifiers on various sub-samples of the dataset in parallel and then combines the trained classifiers to improve predictive accuracy and control over-fitting. In a Gradient Boosting model, decision trees are trained consecutively in a forward stage-wise fashion, where each new tree is fitted to the predecessor's (pseudo) residual error, allowing sequential optimization of an arbitrary differentiable cost function through gradient descent. Artificial neural networks (ANNs) are computing systems characterized by elementary units (called neurons) interconnected through edges with adjustable weights. Such neurons are organized in layers that perform different types of mathematical transformations at their inputs. Typically, the weights of a neural network are adjusted through variants of the gradient descent algorithm, with gradients computed using the backpropagation algorithm. A multi-layer perceptron (MLP) is an ANN with multiple layers between the input and output layers. The MLP used in the study has a basic architecture of 2 fully connected layers with 100 neurons and ReLu activation functions. Adam optimizer (Kingma and Ba, 2014) was used to optimize the weight parameters.

Training of all the models mentioned above was implemented in Python 3.9.7, with scikit-learn 1.0.1 and LightGBM 3.3.1 libraries.

### 2.6 Statistical analysis

To evaluate statistically significant differences between the performance of the models, we compared their AUROC scores by running a statistical test. To statistically compare the performance of a pair of models, we used the Nadeau and Bengio's corrected *t*-test (Nadeau, 2003). This test takes into account the non-independence of the 100 AUROC scores of the individual models, obtained by evaluating the models on the same folds with repeated 10-times 10-fold stratified cross-validation. Finally, for pairwise comparison of all models, we ran the same statistical test multiple times by applying a Bonferroni correction to the computation of the p-values. The significance level was set to p < 0.05.

### 2.7 Feature selection

Initially, the models are trained using all the 1402 input features, and the best learning algorithm is selected for further analysis. Indeed, the overall objective of this work is to build a model as accurate and interpretable as possible. Thus, it was necessary to select a small subset of features sufficiently informative to have an accuracy comparable to the one achieved by using all the 1402 input features. Furthermore, in order to increase interpretability and prevent underestimation of the relative importance of features due to multicollinearity (Gregorutti et al., 2013) the selected features should be ideally uncorrelated or weakly correlated to each other. To achieve this, we used sequential feature selection combined with hierarchical clustering (Murtagh and Contreras, 2012) on some features’ correlation index. In this work, we used the Spearman rank-order index to take into account non-linear relationships between pairs of features. This allowed us to construct a small subset of uncorrelated or weakly correlated features by choosing a given number of clusters and keeping a single feature from each cluster.

Different strategies can be used to select one representative feature from a particular cluster, either through automated methods or domain expert knowledge. In this work, we used an automated strategy. For each cluster, features have been ranked according to their univariate predictivity of the target variable and the most predictive one was picked. The predictivity of a feature was estimated with a two-sample Kolmogorov – Smirnov test (Justel et al., 1997) which empirically measures the distance between the two distribution functions of the considered feature, one referring to sweet and the other referring to bitter instances. The greater the distance between these two empirical distributions, the greater the probability that the sweet and bitter samples are drawn from different distributions, and the greater the univariate capability of the considered feature in predicting the target variable.

### 2.8 Feature importance analysis

To measure and rank the importance of each variable and explain their contribution to the individual predictions of the best performing model, we used SHAP (SHapley Additive exPlanations) values (Lundberg and Lee, 2017), a recent model-agnostic explanation methodology with a solid theoretical foundation and desirable properties. The SHAP explanation method computes Shapley values from the coalitional game theory conceptualized by the economist Lloyd Shapley, hence the name. The feature values of a sample act as players in a coalition and Shapley values tell us how to fairly distribute the resulted prediction among the features. An important feature is that the Shapley values are calculated as an addictive feature attribution method. For machine learning models, this means that SHAP values of all the input features will always sum up to the difference between baseline (expected) model output and the current model output for the prediction being explained. Furthermore, SHAP values are consistent, which means that features that are unambiguously more important are guaranteed to have a higher SHAP value. Operationally, for a single instance x, given a model f that outputs a prediction value $\hat{y}$, SHAP decomposes this prediction into the sum of a baseline value with the contributions that each feature has to the prediction, that is:

(1) $$\hat{y}=y_{base}+\varphi \left(x_{1}\right)+\varphi \left(x_{2}\right)+\varphi \left(x_{3}\right)+\ldots$$

where $y_{base}=E\left[f\left(X\right)\right]$ is the expected value of the predictions of all the training data X and $\varphi \left(x_{j}\right)$ is the SHAP value corresponding to the j-th feature. In our study, positive SHAP values $\varphi \left(x_{j}\right)>0$ implies a positive contribution to the sweetness of the molecule, while negative SHAP values imply a positive contribution to the bitterness of the molecule. $\left|\varphi \left(x_{j}\right)\right|$ gives the magnitude of the contribution. The specific formula for the calculation of $\varphi \left(x_{j}\right)$ is given by the following expression:

(2) $$\varphi \left(x_{j}\right)=\sum_{S\subset N\setminus \left\{j\right\}}\frac{\left|S\right|!\left(M-\left|S\right|-1\right)!}{M!}\left[f_{x}\left(S\cup \left\{j\right\}\right)-f_{x}\left(S\right)\right]$$

where N is the set of all input features with M its dimension, S is a subset of N of dimension |S|, $f_{x}\left(S\right)=E\left[f\left(X\right)|X^{S}=x^{S}\right]$ is the expected value of the predictions conditioned on the subset S of input features with known values $x^{S}$ and $f_{x}\left(S\cup \left\{j\right\}\right)$ is the same but with feature j added to subset S. Finally, the SHAP value for feature j is computed as a weighted average over all possible feature subsets S that don't include feature j already.

A comparison between the different models investigating the bitter/sweet dichotomy is reported in Table S2, highlighting the sources used for the construction of the dataset, the employed molecular descriptors for features computing, and the methods/approaches used for features selection, model building and model interpretation.

### 2.9 Applicability domain

In the current work, we developed an applicability domain (AD) to provide additional information about prediction reliability. An average-similarity approach already employed in previous recent literature in the taste prediction field (Zheng et al., 2018, 2019) was considered. The AD was created considering a random 90:10 dataset partitioning into training and validation sets according to the 10-fold cross-validation employed in the model development. (i) the Morgan Fingerprints (1024 bits, radius 2) were calculated using RDKit for all the compounds in the dataset set; (ii) a similarity score was then evaluated between each molecule in the training and validation sets and the previously-defined fingerprints using the Tanimoto similarity index from RDKit; (iii) then the average similarity score was computed by averaging the similarity scores of the 5 most similar couple of compounds. The distribution of the average similarity scores for the training and validation sets was used to identify a similarity threshold to discriminate between query compounds inside or outside the domain of applicability.

## 3 Results and discussion

### 3.1 Data preprocessing and missing values handling

Different strategies for data preprocessing and imputation of missing values were used according to the different learning models employed. For logistic regression, k-nearest neighbours and multi-layer perceptron the outliers were treated with 90% winsorization, i.e., each variable was clipped at its 5th and 95th percentile, then each feature was scaled with min-max normalization. For gradient boosting and random forest, only 90% of winsorization was applied. The missing values were particularly severe for the molecular distance edge descriptors and the atom type e-state descriptors, respectively 60.8% and 40.4% of missing values on average between the descriptors. These were generated due to chemical or structural characteristics of the molecule that makes the computation of a particular descriptor not possible, thus resulting in *missing not at random* (MNAR) values. For this reason, missing values have been imputed with a constant out-of-distribution value, namely: 1 for logistic regression, k-nearest neighbours and multi-layer perceptron; and -99999 for the random forest. For gradient boosting the missing values were automatically handled by the LightGBM implementation.

### 3.2 Model performances

**Fig. 1:**
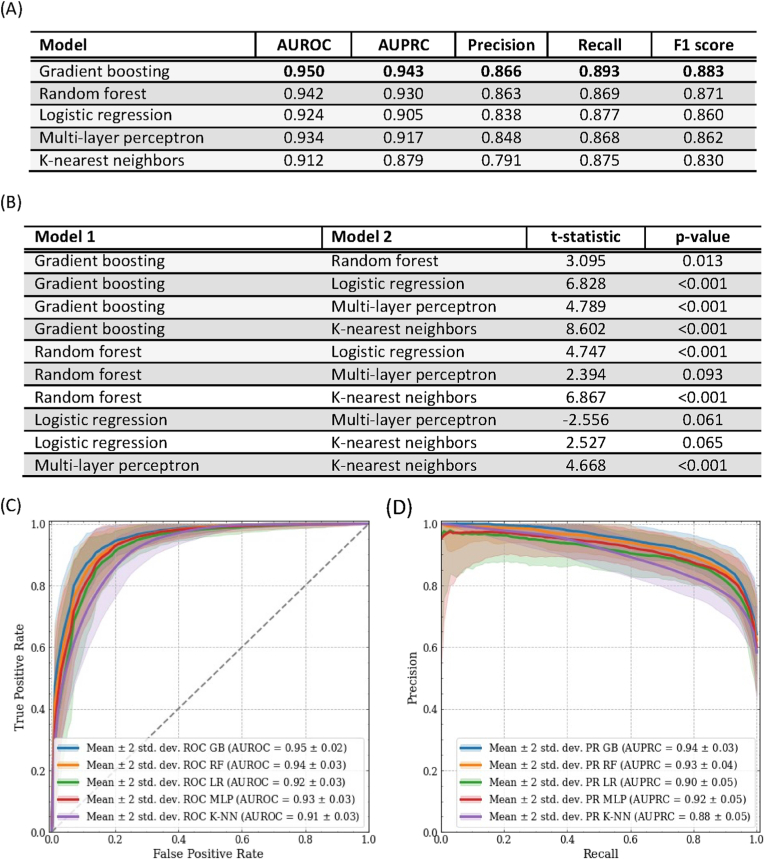
(A) Average model performance. (B) Pairwise comparison of all model performance with Nadeau and Bengio's corrected *t*-test and Bonferroni correction. (C) Solid lines and shaded areas represent the average receiver operating characteristics curves and their 95% confidence intervals. (D) Solid lines and shaded areas represent the average precision-recall curves and their 95% confidence intervals. Abbreviations: GB, gradient boosting, RF, random forest, LR, logistic regression, MLP, multi-layer perceptron, K-NN, k-nearest neighbours.

The complete performances of the tested models, computed with repeated 10-times 10-fold stratified cross-validation and averaged on the folds, are summarized in Fig. 1A–B, and the *receiver operating characteristic* (ROC) curve and *precision-recall* (PR) curve are shown in Fig. 1C–D. Gradient boosting achieved an AUROC of 0.950 (95% CI [0.930, 0.970]); random forest achieved an AUROC of 0.942 (95% CI [0.916, 0.968]); MPL achieved an AUROC of 0.934 (95% CI [0.906, 0.962]); logistic regression and k-nearest neighbours classifier achieved an AUROC of 0.924 (95% CI [0.894, 0.954]) and (95% CI [0.880, 0.944]), respectively.

A direct comparison between our approach and the literature in this field is not completely fair, as performance evaluation is not performed on the same testing data. However, we provide in the following useful overview to contextualize the results achieved by our approach compared to the ones available in the literature and to give an indication of the performance achievable for this type of classification problem. BitterSweetForest (Banerjee and Preissner, 2018) achieved higher metrics (AUROC = 0.98, F1 = 0.92–0.95, ACC = 0.97), but with a remarkably lower number of samples in the database (517 artificial and natural sweet compounds and 685 bitter molecules), limiting the exploration of the bitter/sweet chemical space. BitterSweet (Tuwani et al., 2019) obtained different performances for the sweet/non-sweet and the bitter/non-bitter predictions. In particular, for the sweet/non-sweet prediction, BitterSweet achieved AUPRC of 0.93, AUROC of 0.85, F1 score of 0.77 and regarding the bitter/non-bitter prediction AUPRC of 0.93, AUROC of 0.88, F1 score of 0.86.

### 3.3 Feature selection

**Fig. 2:**
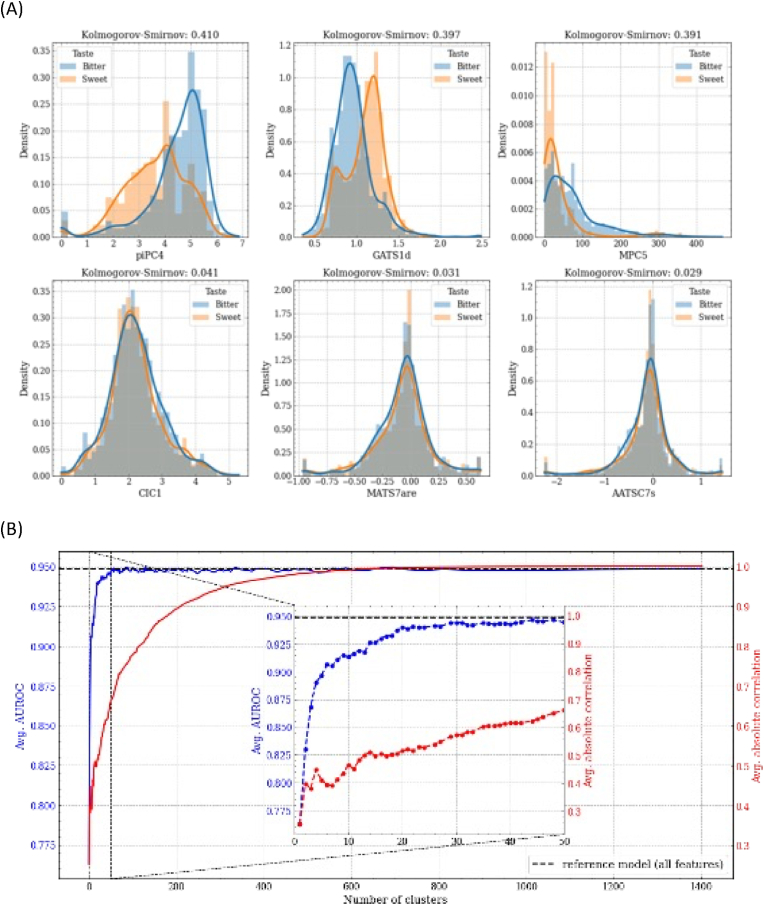
(A) Kernel density estimation of the sweet vs bitter molecules empirical distributions for features with high Kolmogorov – Smirnov statistic (first row) and low Kolmogorov – Smirnov statistic (last row). (B) Feature selection algorithm results. Average AUROC values (blue left y-axis) and average absolute intra-cluster correlation (red right y-axis) as the number of clusters increases. The zoom represents the progress of the algorithm until the first 50 clusters are reached. (For interpretation of the references to colour in this figure legend, the reader is referred to the Web version of this article.)
ii. After converting the correlation matrix to a distance matrix, hierarchical clustering using Ward's linkage was performed and two clusters were selected. From these, the 2 most representative features were picked based on their estimated predictive capacity and used to train a LightGBM model and compute cross-validation performances, together with the intra-cluster mean absolute correlation of the features.
iii. The process described in step (ii) is repeated for 3, 4 clusters, until each cluster is atomic i.e., it contained a single feature.

In order to select a small subset of uncorrelated or weakly correlated informative features, we used sequential feature selection combined with hierarchical clustering on the feature's Spearman rank-order correlations, as described in the following steps.

i. First, the feature correlation matrix was constructed using Spearman rank-order correlations and, for each feature, the predictive capacity of the target variable was estimated through a two-sample Kolmogorov – Smirnov test. In Fig. 2A (first line), the variables piPC4 (conventional bond order ID number of order 4), GATS1d (Geary autocorrelation coefficient of lag 1 weighted by sigma electrons) and MPC5 (molecular path count of order 5) are shown, characterized by high values of the Kolmogorov – Smirnov statistic and high separation between the empirical distributions of samples with sweet target and samples with the bitter target. The second line of the same figure shows the variables CIC1 (1-ordered complementary information content), MATS7are (Moran autocorrelation coefficient of lag 7 weighted), and AATSC7s (Broto autocorrelation of lag 7 weighted by intrinsic state) characterized by low Kolmogorov – Smirnov statistic values and high overlap between the empirical distributions of sweet and bitter samples. The variable with the highest estimated predictive capacity (piPC4) was selected and used to train a LightGBM model. The resulting performances were computed with 5-fold cross-validation and stored.

The results of this procedure are shown in Fig. 2B, where we can observe that with a limited number of features it is still possible to approach the performance of the reference model trained with the entire set of features, which reinforces the fact that groups of features are redundant.

Finally, we have arbitrarily chosen 29 features as a good compromise between model performance (AUROC = 0.944), simplicity and interpretability. Fig. S3 shows the correlation matrix of the selected features and the absolute values of the feature's Spearman rank-order correlations with an average absolute correlation between the variables of 0.19. This shows that the implemented procedure allowed us to develop a model with weakly correlated features as inputs.

### 3.4 Global interpretation

**Fig. 3:**
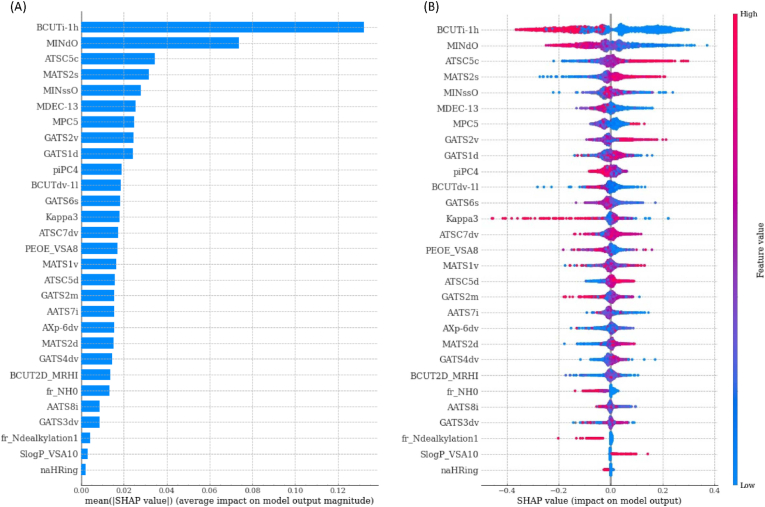
SHAP feature importance plots. (A) The left bar plot represents a ranking of the importance of the variables with their average impact on model prediction. (B) The right dot plot represents each data point with the signed contribution of each variable to the model prediction: blue colour indicates low values for a variable whereas red colour indicates high values. (For interpretation of the references to colour in this figure legend, the reader is referred to the Web version of this article.)

The SHAP explanation method aims to explain the prediction of a single instance by estimating, for each feature, its contribution to the prediction, called SHAP value (in this study the prediction associated with a molecule corresponds to the probability predicted by the model that molecule is sweet). By combining SHAP values computed for each sample of the dataset, we obtain a matrix with one row per sample and one column per feature. From the analysis of this matrix, it is possible to obtain global explanations of the entire model. The SHAP feature importance bar plot shown in Fig. 3A reports the features in descending order of importance computed as the average across the data of the absolute SHAP values. BCUTi-1h (first highest eigenvalue of Burden matrix weighted by ionization potential) and MINdO (the minimum value of the atom type E-state descriptor (Hall and Kier, 1995) linked to the presence of the atom group double bonded with Oxygen) have been identified as the most impacting features, followed by ATSC5c (centred Moreau-Broto autocorrelation of lag 5 weighted by gasteiger charge), MATS2s (Moran autocorrelation coefficient of lag 2 weighted by intrinsic state), MINssO (the minimum value of the atom type E-state descriptor (Hall and Kier, 1995) linked to the presence of the -O- atom group, MDEC- 13 (molecular distance edge between all primary and tertiary carbons), MPC5 (molecular path count of order 5), GATS2v (Geary autocorrelation of lag 2 weighted by van der Waals volumes) and GATS1d (Geary autocorrelation coefficient of lag 1 weighted by sigma electrons). All other features were considered less impacting on predictions. In the SHAP summary plot of Fig. 3B, in which each sample is depicted as a point where the position on the x-axis represents the impact on the prediction in the form of SHAP value and the colour represents the intensity (blue for low values to red for high values) of the value assumed by a feature, feature importance is combined with the directional relationship between values assumed by a feature and impact on predictions. Among the most impacting variables, ATSC5c, MATS2s and GATS2v are positively correlated with the sweetness of a molecule, while BCUTi-1h and MINdO are positively correlated with the bitterness of a molecule.

**Fig. 4:**
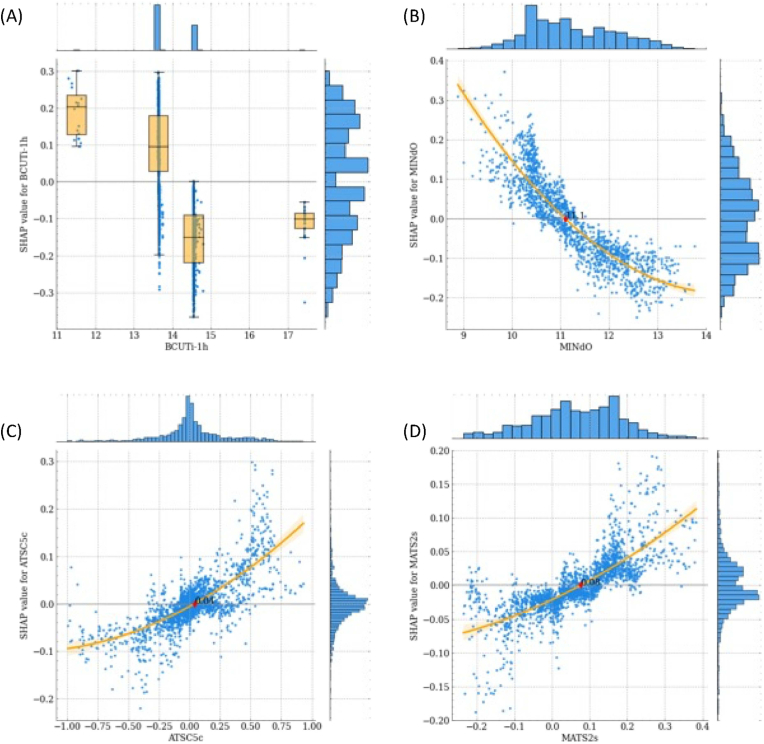
SHAP dependence plots of the 4 most representative features. (A) BCUTi-1h, (B) MINdO, (C) ATSC5c, (D) MATS2s. For discrete and mixed variables, values are plotted with a scatter plot and box plots with whiskers enclosing points belonging to different levels (A). For continuous variables, values are plotted with a scatter plot and an orange regression line with shaded 95% confidence intervals (B, C, D). A red diamond marks a cut-off point of the feature. Empirical distributions of feature and SHAP values are represented with histograms on the top and right of each plot. (For interpretation of the references to colour in this figure legend, the reader is referred to the Web version of this article.)

The empirical form of the relationship between feature values and impact on model predictions can be studied for each feature with the SHAP dependence plots, where each data instance is represented by a point with a position on the x-axis the value assumed by the feature and the position on the y-axis the corresponding Shapley value. The SHAP dependence plots for the 4 most representative features are presented in Fig. 4.

**Fig. 5:**
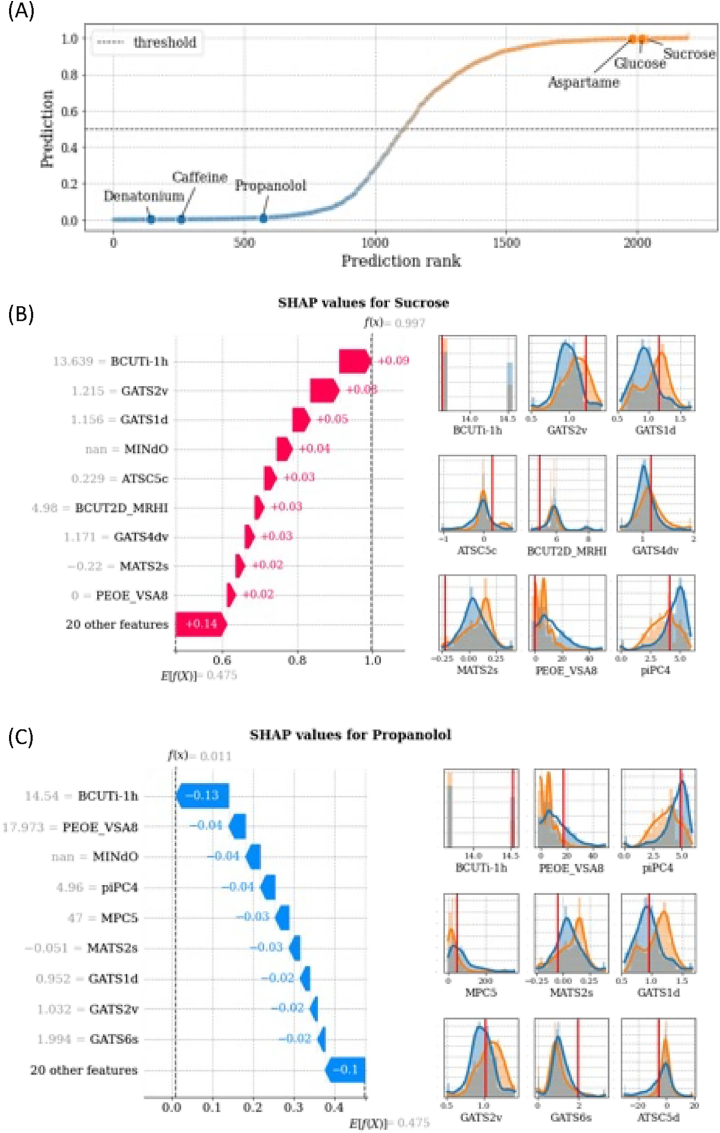
Prediction rank for the molecules of the entire dataset (x-axis) vs out-of-sample predicted sweetness probability (y-axis). Reference molecule prediction are highlighted. SHAP profiles of two representative molecules: Sucrose (B) and Propanolol (C). For each figure, SHAP values are shown in the left panel and impacting feature distributions in the right panel, with values assumed by the features highlighted with solid red lines. (For interpretation of the references to colour in this figure legend, the reader is referred to the Web version of this article.)

### 3.5 Local interpretation

Finally, in this paragraph, we report a local interpretative analysis of the final model using as case studies six representative molecules (Fig. 5 and Fig. S5):

• Three sweet molecules, i.e., Sucrose, Glucose, and Aspartame;
• Three bitter molecules, i.e., Propanolol, Caffein, and Denatonium.

Fig. 5A shows the out-of-sample predictions of the entire dataset obtained in cross-validation and ordered according to the prediction ranking. The considered six reference molecules are highlighted in the plot with their sweet/bitter target correctly predicted by the model.

The SHAP profiles of the representative molecules are shown in the left panel of Fig. 5B–C and Figs. S5A–D. The most impacting features for the prediction are shown on the y-axis and the corresponding SHAP values are displayed through coloured arrows with their cumulative value reported on the x-axis. Positive SHAP values, represented with red arrows, indicate a positive contribution to the predicted sweetness of the molecule, while negative SHAP values, represented with blue arrows, indicate a positive contribution to the predicted bitterness of the molecule. Empirical distributions of the most impacting features are reported in the right panels of Fig. 5B–C and Figs. S5A–D. The orange colour distributions correspond to the sweet molecules in the dataset, while the blue ones correspond to the bitter molecules. The vertical solid red lines highlight the value assumed by the feature in the corresponding molecule. If the value is missing, the feature is skipped. For these molecules, the most impacting features contribute with the same sign to the prediction. Moreover, the most impacting feature is unanimously BCUTi-1h (first highest eigenvalue of Burden matrix weighted by ionization potential). For bitter molecules, other common impacting features are MINdO (the minimum value of the atom type E-state descriptor (Hall and Kier, 1995) linked to the presence of the atom group double bonded with Oxygen) and MPC5 (molecular path count of order 5), while for sweet molecules they are GATS2v (Geary autocorrelation of lag 2 weighted by van der Waals volumes) and GATS1d (Geary autocorrelation coefficient of lag 1 weighted by sigma electrons).

## 4 conclusion

The sweet-bitter dichotomy is an extremely fascinating aspect of taste perception: while the sweet taste is commonly associated with a pleasant sensation linked to the energetic content of foods, bitter is a complex control system normally related to the ability to avoid toxic or possibly harmful substances. In this work, we have further investigated this attractive mechanism to shed light on the molecular features determining the taste of a specific molecule. Therefore, we developed a machine-learning-based classifier able to discriminate between the bitter and sweet tastes of a query compound based on its molecular structure. The implemented tool is based on the widely used SMILES representation and employs open-source molecular descriptors to calculate the features on which the model relies. Thanks to statistical analysis methods, feature selection and analysis techniques, we were able to pinpoint a reduced number of molecular features determining the bitter or sweet taste and, together with the SHAP explainability method, we underlined the impact of the selected features, providing an informed and interpretable classification. In the process of designing new molecules, it is difficult to make use of the selected features as they are not intuitive. This issue, however, is not related to the selection of features, but rather to the use of molecular features in 2D. This point could be adequately addressed not only by simplifying the input molecular features, which will inevitably reduce algorithm performance, but also by taking advantage of a number of scientific studies focused on machine learning decoders able to reconstruct the chemical information starting from the 2D features of the molecule. Additionally, a generative model could be added to the computational pipeline to suggest appropriate chemical changes to achieve the desired taste. Addressing the previously-mentioned challenges represent the future development of this work, and we hope that our study will provide a starting point for potential studies in this field. The developed model will therefore pave the way toward the rational design and screening of sweet/bitter molecules through the molecular understanding of the physical and chemical characteristics underlying the perception of these tastes. To ensure the reproducibility of the results and to allow the usage of the developed model, we publicly release the Python scripts, along with the employed datasets and supplementary material on GitHub (https://github.com/gabribg88/VirtuousSweetBitter). The sweet/bitter classifier will be also implemented into a user-friendly webserver to allow its usage even to non-expert or technical users. In a broader view, this tool will be integrated into the framework of an EU-funded project, named VIRTUOUS (64), which aims at creating an intelligent computational platform by integrating molecular modelling methods, drug discovery techniques, machine learning classifiers, algorithms for big data, cloud computing, and experimental data to predict the organoleptic profile of selected types of food based on their chemical composition. In conclusion, the present work represents a crucial starting point in the definition of a *virtual tongue* able to predict the taste of specific ingredients and general compounds with the ultimate goal of shedding light on the mechanisms and hidden relationships at the basis of the taste perception process.

## Author contributions

Conceptualization: G.M., L.P., M.A.D, D.P. and G.G.; Model development: G.M. and L.P.; Data Curation: L.P. and G.M.; Supervision: G.G, D.P., and M.A.D.; Coordination: G.G., D.P. and M.A.D; Project Administration: G.G. and M.A.D.; Funding Acquisition: M.A.D. All authors wrote the paper and critically commented on the manuscript. All authors read and approved the final manuscript.

## Declaration of competing interest

No competing interest to be declared.

## Data Availability

The data and code of this study are available at the GitHub link indicated in the manuscript
