# Supplementary material for: Informed classification of sweeteners/bitterants compounds via explainable machine learning
Source: Curr Res Food Sci. 2022 Nov 14;5:2270–80. doi: 10.1016/j.crfs.2022.11.014 (PMC9682350; doi:10.1016/j.crfs.2022.11.014)
Supplement: Multimedia component 1 [file mmc1.pdf]

Supplementary Material

Informed Classification of Sweeteners/Bitterants Compounds via Explainable Machine Learning

Gabriele Maroni[1], Lorenzo Pallante[2], Giacomo Di Benedetto[3], Marco A. Deriu[2], Dario Piga[1], Gianvito Grasso[1*]

[1] Dalle Molle Institute for Artificial Intelligence IDSIA - USI/SUPSI, Via la Santa 1, CH-6962 Lugano-Viganello, Switzerland

[2] Polito[BIO]MedLab, Department of Mechanical and Aerospace Engineering, Politecnico di Torino, Corso Duca degli Abruzzi 24, 10129, Torino, Italy.

[3] 7HC srl, Via Giovanni Paisiello 55, 00198, Rome, Italy

Table S1. Summary of the collected compounds from the selected taste databases.

Reference	Taste	Number
**Biochemical Targets of Plant Bioactive Compounds by Gideon Polya**(Polya, 2003)	Bitter	39
	Sweet	32
**BitterDB**(Wiener et al., 2012)	Bitter	1018
**Fenaroli Handbook of Flavor Ingredient**(Burdock, 2016)	Bitter	16
	Sweet	419
**Rodgers et al. (2006)** (Rodgers et al., 2006)	Bitter	17
**Rojas et al. (2017)**(Rojas et al., 2017)	Bitter	69
	Sweet	427
**SuperSweet**(Ahmed et al., 2011)	Sweet	265
**The Good Scents Company Database**	Bitter	37
	Sweet	153
**Wiener et al. (2017)**(Dagan-Wiener et al., 2017)	Bitter	75
**SweetenersDB**(Chéron et al., 2017)	Sweet	119

Table S2. Comparison of the main bitter/sweet prediction models

Reference	***Source***	***Molecular descriptors***	***Feature selection***	***(Best) Modelling approach***	***Interpretation***
BitterSweetForest(Banerjee & Preissner, 2018)	BitterDB and SuperSweet	Morgan, Atom-Pair, Torsion and Morgan Feat fingerprints from RDkit	Based on performance	Random Forest with Morgan fingerprint	Bayesian-based feature analysis
BitterSweet(Tuwani et al., 2019)	Biochemical Targets of Plant Bioactive Compounds by<br><br>Gideon Polya, BitterDB, SuperSweet, Fenaroli's Handbook of Flavor Ingredients (5th Edition), Rodgers et al., Rojas et al.,	ChemoPy, Dragon 2D, Dragon 2D/3D, Canvas and ECFPs	Boruta feature selection algorithm and PCA	Dragon2D/3D<br><br>molecular descriptor and Boruta feature selection with Adaboost	Random forest relative feature importance with mean decrease in Gini impurity

	TOXNET, The Good Scents Company Database, Wiener et al.			(sweet/non-sweet), Dragon2D/3D molecular descriptor and PCA with Adaboost (bitter/non-bitter)	
VirtualTaste(Fritz et al., 2021)	BitterDB, SuperSweet and BitterSweetForest tool	MACCS and Morgan fingerprints from RDkit	\	Random Forest	Bayesian-based feature analysis
Ours	Biochemical Targets of Plant Bioactive Compounds by Gideon Polya, BitterDB, SuperSweet, Fenaroli's Handbook of Flavor Ingredients (5th Edition), Rodgers et al., Rojas et al., The Good Scents Company Database, Wiener et al., SweetenersDB	2059 molecular descriptors from RDkit, pybel and Mordred open-source libraries	Sequential feature selection based on hierarchical clustering on the feature's Spearman rank-order and two-sample Kolmogorov - Smirnov test	Gradient Boosting (LightGBM)	Global (feature importance, dependence plots) and local interpretation (features impact on individual predictions) based on SHAP values

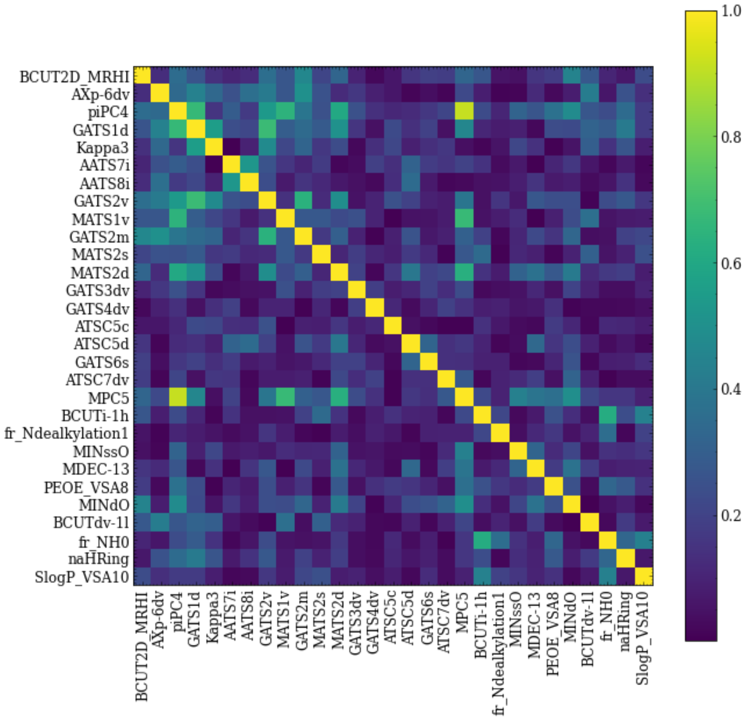

Figure S3. Heatmap of the selected features correlation matrix computed with Spearman's rank correlation in absolute value.

Validation on non-bitter/non-sweet molecules

This study is focused on the sweet/bitter dichotomy in order to isolate the most suitable variables capable of highlighting the differences between sweet and bitter compounds. However, it is also interesting to analyze the behavior of the model, fed only by the variables selected in this work, in the prediction of neither sweet nor bitter molecules. For this purpose, the original training dataset consisting of 2686 compounds (1415 sweet and 1271 bitter) was augmented by 198 additional compounds classified in the literature as neither bitter nor sweet(Burdock, 2016; Mullard, 2017; Rojas et al., 2017) by converting the original binary

classification problem into a multiclass problem with 3 labels. The final LightGBM model was retrained on this augmented dataset and performance was assessed according to a stratified 5-fold cross-validation strategy. For each class, the ROC curves are computed through a one-vs-rest method (namely, performance of the considered class against the remaining two ones) and shown in Figure S4, along with the macro-average ROC curve, which equally weights each point of the single ROC curve. Also, for this 3-class problem, the predictive performance is satisfactory, with an average AUROC equal to 0.92. Note that the AUROC for the Non-bitter/sweet class is slightly worse than the average (0.89). This was expected since the features used by the predictive model was chosen only considering bitter and sweet compounds.

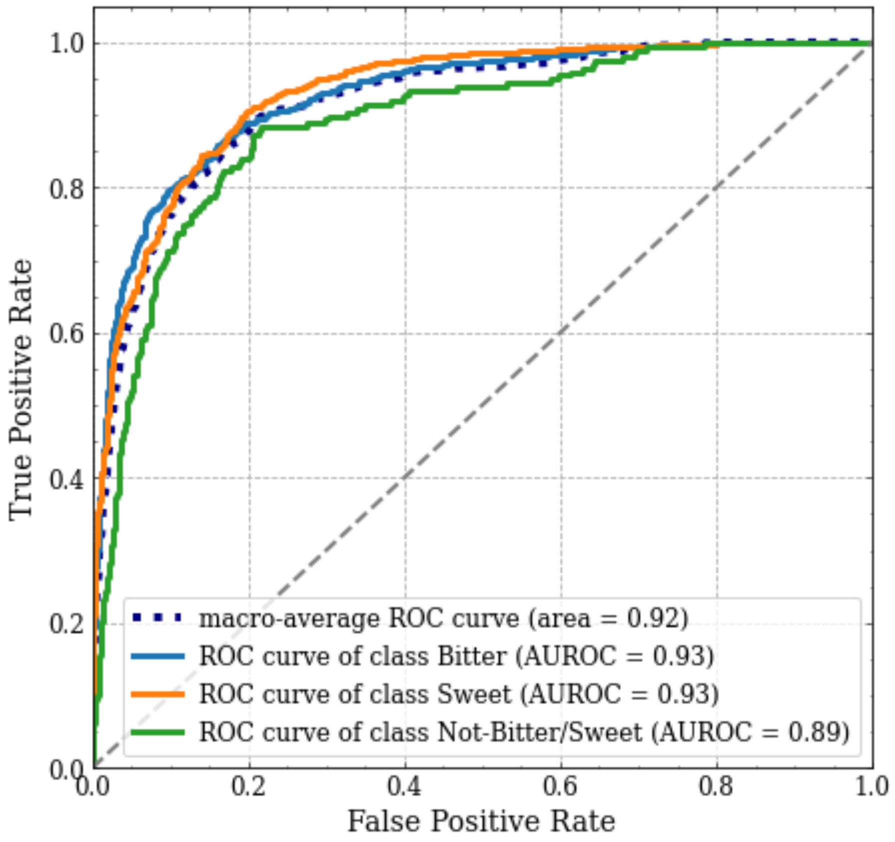

Figure S4. One-vs-rest ROC curves: bitter vs others (blue); sweet vs others (orange); not bitter/not-sweet vs others (green); macro-average ROC curves (dotted dark blue).

Local interpretation

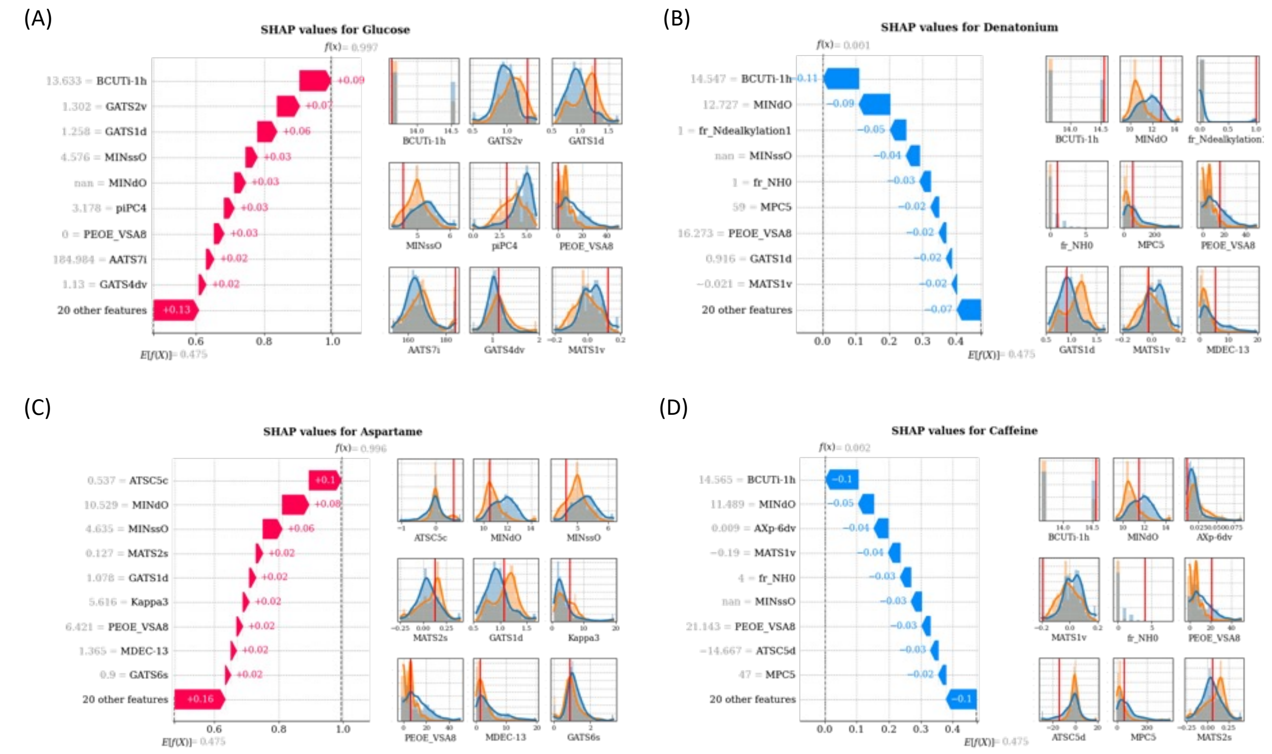

Figure S5. SHAP profiles of four representative molecules: Glucose (A), Denatonium (B), Aspartame (C) , and Caffeine (D). For each figure, SHAP values are shown in the left panel and impacting feature distributions in the right panel, with values assumed by the features highlighted with solid red lines.

BIBLIOGRAPHY

Ahmed, J., Preissner, S., Dunkel, M., Worth, C. L., Eckert, A., & Preissner, R. (2011). SuperSweet—A resource on natural and artificial sweetening agents. *Nucleic Acids Research*, *39*(Database), D377–D382. https://doi.org/10.1093/nar/gkq917

Banerjee, P., & Preissner, R. (2018). BitterSweetForest: A Random Forest Based Binary Classifier to Predict Bitterness and Sweetness of Chemical Compounds. *Frontiers in Chemistry*, *6*, 93. https://doi.org/10.3389/fchem.2018.00093

Burdock, G. A. (2016). *Fenaroli's Handbook of Flavor Ingredients* (0 ed.). CRC Press. https://doi.org/10.1201/9781439847503

Chéron, J.-B., Casciuc, I., Golebiowski, J., Antonczak, S., & Fiorucci, S. (2017). Sweetness prediction of natural compounds. *Food Chemistry*, *221*, 1421–1425. https://doi.org/10.1016/j.foodchem.2016.10.145

Dagan-Wiener, A., Nissim, I., Ben Abu, N., Borgonovo, G., Bassoli, A., & Niv, M. Y. (2017). Bitter or not? BitterPredict, a tool for predicting taste from chemical structure. *Scientific Reports*, *7*(1), 12074. https://doi.org/10.1038/s41598-017-12359-7

Fritz, F., Preissner, R., & Banerjee, P. (2021). VirtualTaste: A web server for the prediction of organoleptic properties of chemical compounds. *Nucleic Acids Research*, *49*(W1), W679–W684.

https://doi.org/10.1093/nar/gkab292

Mullard, A. (2017). The drug-maker's guide to the galaxy. *Nature*, *549*(7673), 445–447. https://doi.org/10.1038/549445a

Polya, G. (2003). *Biochemical Targets of Plant Bioactive Compounds: A Pharmacological Reference Guide to Sites of Action and Biological Effects* (0 ed.). CRC Press. https://doi.org/10.1201/9780203013717

Rodgers, S., Glen, R. C., & Bender, A. (2006). Characterizing Bitterness: Identification of Key Structural Features and Development of a Classification Model. *Journal of Chemical Information and Modeling*, *46*(2), 569–576. https://doi.org/10.1021/ci0504418

Rojas, C., Todeschini, R., Ballabio, D., Mauri, A., Consonni, V., Tripaldi, P., & Grisoni, F. (2017). A QSTR-Based Expert System to Predict Sweetness of Molecules. *Frontiers in Chemistry*, *5*, 53. https://doi.org/10.3389/fchem.2017.00053

Tuwani, R., Wadhwa, S., & Bagler, G. (2019). BitterSweet: Building machine learning models for predicting the bitter and sweet taste of small molecules. *Scientific Reports*, *9*(1), 7155. https://doi.org/10.1038/s41598-019-43664-y

Wiener, A., Shudler, M., Levit, A., & Niv, M. Y. (2012). BitterDB: A database of bitter compounds. *Nucleic Acids Research*, *40*(D1), D413–D419. https://doi.org/10.1093/nar/gkr755
